# An Editorial Introduction: A Vision for *eJHaem*

**DOI:** 10.1002/jha2.70004

**Published:** 2025-02-26

**Authors:** Phillip Scheinberg

**Affiliations:** ^1^ Division of Haematology Hospital A Beneficência Portuguesa São Paulo Brazil

It is with a great sense of privilege that I assume the role of Editor‐in‐Chief of *eJHaem*, the open‐access journal of the British Society for Haematology (BSH). Established in 2020, *eJHaem* is a relatively young journal that has already become an important publication within the BSH family of journals.

Since its inception, Dr. Andrew Evens has brilliantly led *eJHaem*. Under his guidance, the Journal has published a wide variety of content, including original articles, short reports, case reports, letters, correspondence, and striking images in haematology. The journal covers all aspects of haematology—both benign and malignant diseases, adult and paediatrics—as well as topics in cell therapy (HSCT and CAR‐T) and transfusion medicine.

As Editor‐in‐Chief, I aim to expand the journal's reach, ensuring it becomes a truly global venue representing physicians, scientists, and researchers from diverse regions. A bit about my background: I was born in Boston and raised in São Paulo, Brazil. I returned to the United States for medical training, where I spent 15 years completing my internship, residency, chief residency, and fellowships in both haematology and oncology. For over a decade, I worked at the Haematology Branch of the National Heart, Lung, and Blood Institute (NHLBI) at the National Institutes of Health (NIH) in Bethesda, Maryland. During my fellowships at the NIH, I conducted research both at the bench and in the clinic. After this period, I returned to São Paulo in 2012 to become the Head of the Division of Haematology at one of the largest cancer centres in the country. My international experience has impacted my vision for *eJHaem*: to make it not only accessible but also truly “author‐friendly” for contributors worldwide, while expanding its reach to less represented regions.

It is bittersweet to step down from my role as an Associate Editor (AE) of the *British Journal of Haematology* (*BJHaem*), where I have served since 2019. I am grateful for the warm welcome I received and for the opportunity to contribute to *BJHaem*. I particularly want to thank Dr. John Barrett, Editor‐in‐Chief during my tenure. With his leadership, talent, and knowledge, John excelled at enhancing the quality and breadth of *BJHaem*’s publications and assembling a truly top‐notch team of Associate Editors. I would also like to express my gratitude to Lorna Wycherley, the journal's exceptional Editorial Assistant, whose efforts kept the editorial process running smoothly, and Claire Dowbekin, Wiley's senior publisher, for her openness to innovation, collaboration, and support.

As I take on this new role, I am excited to continue working alongside Dr. Andrew Evens, who is stepping into the role of Editor‐in‐Chief at *BJHaem*. Andy has outlined a clear and inspiring vision for *BJHaem*, which I fully endorse for *eJHaem* [1]. His directives include streamlining the editorial process, fostering global partnerships with leading researchers and institutions, enriching content, expanding global impact, and ensuring the timely dissemination of emerging trends. He also emphasizes the importance of translational science, health economics, equity, and leveraging digital technologies to connect professionals with cutting‐edge advancements in research and clinical practice.

The recent inclusion of *eJHaem* in the Web of Science and PubMed will further enhance its visibility, credibility, and academic reputation, positioning it on the path to receiving its first impact factor later this year. I am excited to collaborate with Andy, Wiley, and the BSH to uphold the leadership of *eJHaem* in haematology, fostering progress and excellence. This partnership will align *eJHaem* with the evolving landscape of research and clinical practice, driving the journal's growth as a premier source of knowledge, innovation, and inspiration for years to come.
